# Hospitalized patients received furosemide undergoing acute kidney injury: the risk and prediction tool

**DOI:** 10.1186/s40001-023-01306-0

**Published:** 2023-09-02

**Authors:** Chen Guan, Chenyu Li, Lingyu Xu, Lin Che, Yanfei Wang, Chengyu Yang, Ningxin Zhang, Zengying Liu, Long Zhao, Bin Zhou, Xiaofei Man, Hong Luan, Yan Xu

**Affiliations:** 1 Department of Nephrology, The Affiliated Hospital of Qingdao University, 16 Jiangsu Road, Qingdao, 266003 China; 2 Medizinische Klinik und Poliklinik IV, Klinikum der Universität, LMU München, Munich, Germany

**Keywords:** Furosemide, Hospital-acquired acute kidney injury, Nomogram, Prediction

## Abstract

**Purpose:**

Furosemide, a frequently prescribed diuretic for managing congestive heart failure and edema, remains a topic of debate regarding its potential risk of inducing acute kidney injury (AKI) in patients. Consequently, this study aims to examine the occurrence of hospital-acquired AKI (HA-AKI) in hospitalized patients who are administered furosemide and to investigate potential risk factors associated with this outcome.

**Methods:**

This study encompassed a cohort of 22374 hospitalized patients who either received furosemide treatment or not from June 1, 2012, to December 31, 2017. Propensity score matching was employed to establish comparability between the two groups regarding covariates. Subsequently, a nomogram was constructed to predict the probability of AKI occurrence among patients who underwent furosemide treatment.

**Results:**

The regression analysis identified the single-day total dose of furosemide as the most significant factor for AKI, followed by ICU administration, estimated glomerular filtration rate, antibiotic, statin, NSAIDs, β-blockers, proton pump inhibitor, chronic kidney disease, and 7 other indicators. Subgroup analysis revealed a synergistic effect of furosemide with surgical operation, previous treatment with β-blockers, ACEI/ARB and antibiotics, leading to an increased risk of AKI when used in combination. Subsequently, a visually represented prognostic nomogram was developed to predict AKI occurrence in furosemide users. The predictive accuracy of the nomogram was assessed through calibration analyses, demonstrating an excellent agreement between the nomogram predictions and the actual likelihood of AKI, with a probability of 77.40%.

**Conclusions:**

Careful consideration of factors such as dosage, concurrent medication use, and renal function of the patient is necessary for clinical practice when using furosemide. Our practical prognostic model for HA-AKI associated with furosemide use can be utilized to assist clinicians in making informed decisions about patient care and treatment.

**Supplementary Information:**

The online version contains supplementary material available at 10.1186/s40001-023-01306-0.Width

## Introduction

Hospital-acquired acute kidney injury (HA-AKI) is a severe medical condition that often occurs in hospitals and is characterized by the deterioration of kidney function, which is associated with an increased risk of mortality [1]. AKI has been reported to occur in approximately 10–15% of hospitalized patients, and the incidence of AKI in intensive care units (ICU) exceeds 50% [2]. Despite advances in diagnostic techniques and renal replacement therapies, patients with AKI are at a high risk of experiencing irreversible acute damage. They may progress to chronic kidney disease (CKD), which increases their risk of developing cardiovascular disease and other complications [3, 4]. The efficacy of current strategies, including early initiation of renal replacement therapy, in improving outcomes for critically ill patients is still debatable [5, 6]. Given the absence of reliable prediction tools, limited active treatment choices, unpredictable prognosis, and the substantial healthcare expenses linked to AKI, it becomes imperative to prioritize the prevention and management of risk factors associated with this condition.

Several risk factors of AKI have been identified, including infection, cardiac insufficiency, hypovolemia, severe trauma, and nephrotoxins, such as aminoglycoside antibiotics, nonsteroidal anti-inflammatory drugs (NSAIDs), and radio-contrast agents [7–9]. Specifically, adverse drug reactions are a common cause of kidney injury [10], with approximately 14% of AKI cases occurring in hospitalized patients and 19% in the ICU being drug-induced [11]. Moreover, it should be noted that a single type of drug may cause a variety of kidney damage [12]. Diuretics are known to be a common cause of AKI among these drugs, accounting for 18.5% of all drug-induced AKI cases, ranking only after systemic antibacterial drugs [13]. As such, the prevention and management of these risk factors remain critical.

Furosemide is one of the most commonly used diuretics for congestive heart failure and edema. It acts by blocking Na–K–Cl_2_ co-transporters on the ascending loop of Henle, resulting in natriuresis and diuresis [14]. In patients with AKI, fluid and electrolyte imbalances are challenging to manage, and clinicians often resort to high doses of furosemide to increase urine output, which may increase the risk of renal injury in reverse [15]. However, the exact relationship between furosemide and AKI is still not fully understood.

The objective of our study is to investigate the current trend of furosemide use in hospitalized patients and to identify the risk factors associated with furosemide use in AKI patients. We also aim to evaluate the different usage methods of furosemide and the associated risk factors in patients with AKI, with the ultimate goal of advancing the prevention and treatment of furosemide-associated renal injury.

## Materials and methods

### Study population

This retrospective study was conducted at a single center over a 6 year period. The study included a total of 430000 in-patients who were admitted between June 1, 2012, and December 31, 2017.

Patients were excluded if they met one of the following characteristics:

1. Age < 18 years (97892 patients)
2. Patients with the latter defined as a diagnostic code for AKI at admission or a change in serum creatinine (Scr) on the first day of admission (1796 patients)
3. Less than one Scr test (96928 patients)
4. More than twice Scr test but > 7 days (39524 patients)
5. ESRD or Kidney transplantation (1087 patients)
6. Missing inpatient data (98753 patients)
7. Hospitalization less than 24 h (41854 patients)

The standard of care was applied to all subjects without any intervention from the study. Clinical data were collected from electronic medical records and databases, and patient anonymity was ensured to protect their privacy. The study was approved by the Institutional Review Board, and the requirement for informed consent was waived.

### Definition and diagnosis

The diagnostic criteria and classification of AKI were based on Kidney Disease: Improving Global Outcomes (KDIGO) 2012 as follows: (1) Scr increased by over 26.5 mmol/L (0.3 mg/dL) within 48 h; or (2) Scr increased to over 1.5-fold of baseline value; or (3) urine output < 0.5 mL/kg/h for more than 6 h. AKI staging was defined according to the KDIGO criteria. AKI diagnosis time is first to reach the KDIGO guide standard. Baseline Scr was defined as the first Scr value measured during hospitalization [16].

To access the association between furosemide and the occurrence of AKI, the analysis was restricted to HA-AKI, excluding patients admitted to the hospital with the diagnosis of AKI or a change of Scr on the first day of admission. HA-AKI was defined as an elevation of Scr after hospitalization exceeding 24 h [17].

Exposure to furosemide was defined based on any filled prescriptions for furosemide before the date of detection of AKI in patients with AKI and prior to the last serum Scr test in patients without AKI. In evaluating the dose-dependent effect of furosemide on the outcomes of AKI patients, furosemide dose was defined as the intravenous administration plus 0.5 × oral dose. The single-day total use of furosemide was presented as milligrams per kilogram daily. The cumulative dosage was added up by the difference in days between the starting and stopping time of each period of consecutive drug usage. The single maximum dose of furosemide was defined as the maximum of all daily doses.

The baseline estimated glomerular filtration rate (eGFR) was calculated using the Chronic Kidney Disease–Epidemiology Collaboration equation [18]. Other patients' comorbidities were defined according to the International Classification of Disease (ICD) 10th Revision (WHO, 1992).

### Data collecting and preprocessing

Approximately 200 variables were collected during the patient's hospitalization. Blood and urine samples were obtained from all patients, and comprehensive blood counts, blood chemistry analyses, and urine tests were conducted on the first day of admission. In addition, essential demographic information such as gender, age, body mass index (BMI), inpatient department (medical, surgical, oncology, gynecology and ICU), concomitant diseases (hypertension, diabetes, coronary heart disease, CKD), and hospitalization-related factors (length of stay, surgery and no. of death) were recorded. Detailed data on furosemide therapy and concomitant medications were also collected, including the single-day total dose, cumulative dose, duration, and other combined drugs.

Variables with missing data exceeding 15% were excluded from the analysis. Multiple imputation was performed using the *mice* package [19], with all model variables considered simultaneously. Given the assumption that the missing data were missing at random [20], we employed the predictive mean matching method [21] to generate five complete imputed data sets that were fitted with logistic models. In addition, continuous variables were transformed into categorical variables based on recognized cut-off values.

### Propensity score matching

To account for measured confounding, we employed 1:1 propensity score matching (PSM) using logistic regression with a caliper of 0.2 of the standard deviation [22]. The PSM matched patients based on selected covariates, considering the exposure to furosemide and stratifying them by baseline characteristics and other variables associated with hospitalization, such as inpatient department, surgery, and concomitant drugs. Standardized mean differences (SMD) and absolute standardized mean difference (ASMD) were used to assess covariate balance after matching between furosemide users and the control group after matching, and an SMD or an ASMD < 0.1 was considered balancing [23].

### Development and verification of the prediction model

We conducted forward stepwise linear regression analysis on patient characteristics and biochemical indices to identify the most significant variables associated with furosemide use in AKI patients and remove multicollinearity. The Akaike information criterion (AIC) was used as the stopping rule [24], with predictor variables having a *P* value < 0.001 and lower AIC being entered into the multivariable logistic models. Then, we used the *Rms* (version 5.1.1) to construct a prognostic nomogram that predicts the risk of AKI in patients [25]. To evaluate the model's discrimination, we measured the concordance index (C-index), which estimates the probability of consistent predicted results with the actual situation [26].

### Statistical analysis

The correlation analyses were performed by performing Pearson’s or Spearman’s rank analysis. The Kaplan–Meier method was used to depict the differences in the daily average dose of furosemide, while the Mantel–Haenszel log-rank test was performed to evaluate the differences in the groups classified by the duration of furosemide. One-way analysis of variance (ANOVA) or Kruskal Wallis test was applied to calculate differences for a variable with more than two categories, followed by Bonferroni post hoc test. The results were compared using the *χ*^2^ test or Fisher’s exact test. The continuous variables were compared by performing *t* tests or Mann–Whitney *U* tests for variables with a non-normal distribution. All analyses were performed using *R* version 3.4.2 (http://www.r-project.org/).

## Results

### Baseline characteristics of the patients

**Fig. 1:**
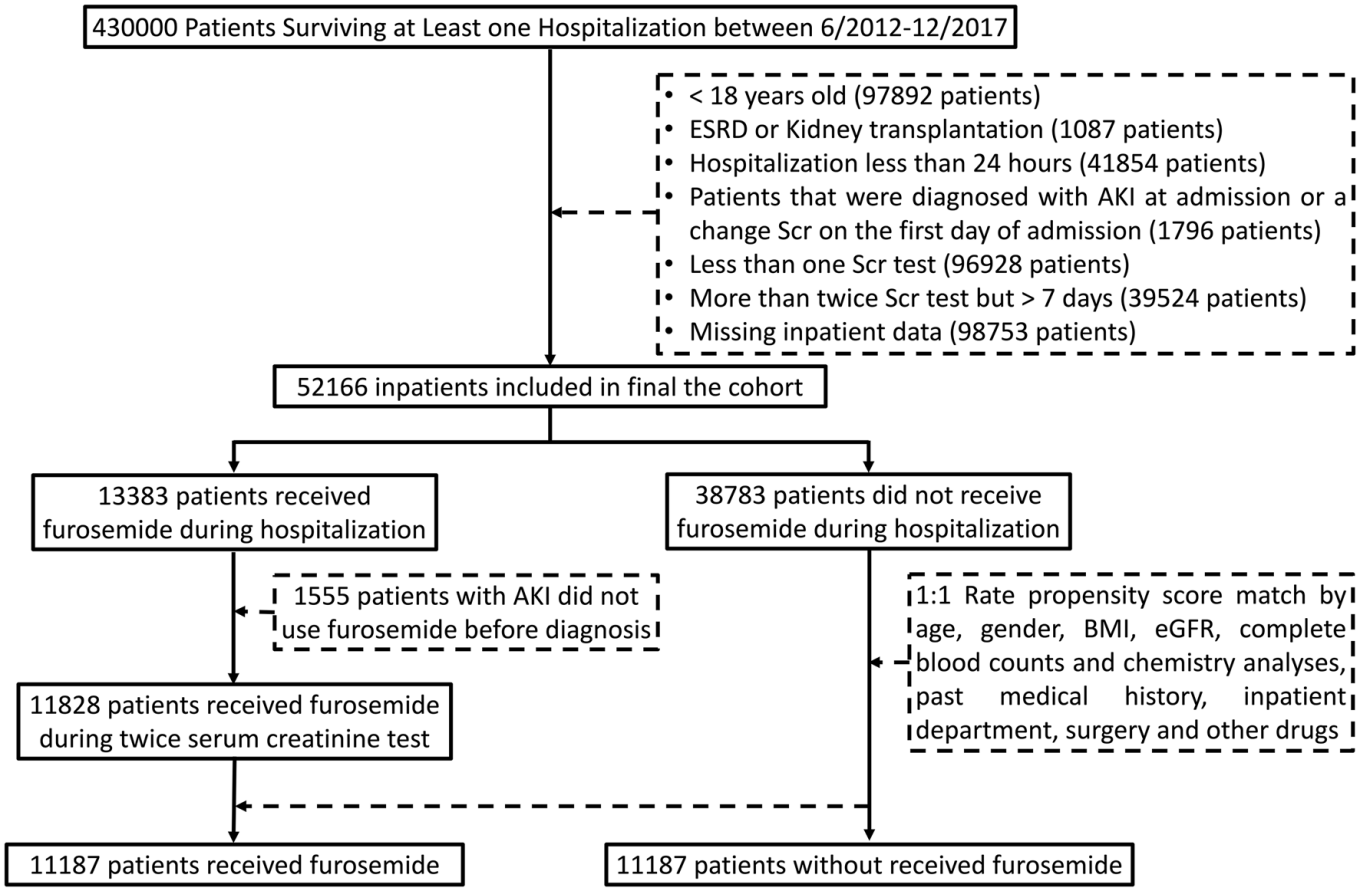
Flow diagram of patient selection

Figure 1 depicts the process of selecting the final cohort analyzed in this study. A total of 11187 pairs of inpatients who either received furosemide or not during hospitalization between June 1, 2012, and December 31, 2017, met the eligibility criteria. Before propensity score weighting, the furosemide group exhibited characteristics, such as older age, more extended hospital stays, lower platelet (PLT) and hemoglobin (Hb) levels, lower eGFR, and a higher proportion of antibiotic use than the control group (Table 1). Furthermore, the incidence of AKI in the furosemide group was 26.88%, whereas it was 10.73% in the control group. These findings indicate that furosemide was more likely to be administrated to patients with severe illness. To mitigate this bias, we applied PSM to establish a more comparable control group. After PSM, the furosemide and control groups showed a well-balanced distribution across most characteristics (*ASMD* < 0.1). However, the incidence of AKI was still higher in the furosemide group than in the control group (23.94% vs. 16.72%, *P* < 0.05, Table 1).

**Table 1:** Baseline characteristics of inpatients taking furosemide or not, before and after propensity score matching

	Propensity score weighting					
	Before			After		
	Furosemide	Control	ASMD^a^	Furosemide	Control	ASMD^a^
Number	11828	38783		11187	11187	
Age	60.59 ± 14.78	57.45 ± 15.12	0.195	60.36 ± 15.13	60.44 ± 14.76	0.0072
Gender	7004 (59.22)	21683 (55.91)	0.0673	6659 (59.52)	6594 (58.94)	0.0118
BMI	24.27 ± 3.84	24.62 ± 3.85	0.0888	24.33 ± 3.87	24.28 ± 3.84	0.0063
Length of stay	12.78 ± 7.38	9.46 ± 5.12	0.4502	11.81 ± 6.79	12.07 ± 6.5	0.0353
AKI	3179 (26.88)	4161 (10.73)		2678 (23.94)	1870 (16.72)	
Stage1	2372 (20.05)	3667 (9.46)		2028 (18.13)	1599 (14.29)	
Stage2	504 (4.26)	311 (0.8)		405 (3.62)	157 (1.4)	
Stage3	303 (2.56)	183 (0.47)		245 (2.19)	114 (1.02)	
PLT	214.47 ± 90.18	221.38 ± 79.89	0.0968	214.89 ± 84.59	216.09 ± 89.77	0.004
Hb	120.84 ± 25.8	127.94 ± 22.63	0.2641	122.03 ± 24.47	121.58 ± 25.59	0.0035
WBC	7.68 ± 3.86	7.36 ± 3.66	0.0753	7.77 ± 4.04	7.63 ± 3.81	0.0093
ALT	45.99 ± 143.61	37.78 ± 101.01	0.048	43.15 ± 118.97	44.55 ± 137.04	0.0027
AST	47.39 ± 157.41	34.92 ± 112.92	0.1318	43.29 ± 147.28	45.56 ± 151.21	0.0046
ALB	34.45 ± 6.93	37.52 ± 6.12	0.4235	35.2 ± 6.47	34.69 ± 6.89	0.0044
UA	326.41 ± 139.71	305.54 ± 114.6	0.1777	316.07 ± 132	323.47 ± 137.38	0.0106
eGFR	90.25 ± 35.78	101.76 ± 33.63	0.2435	98.90 ± 34.40	99.24 ± 34.57	0.0086
CKD	1312 (11.09)	1440 (3.71)	0.235	964 (8.62)	1064 (9.51)	0.0285
DM	2181 (18.44)	4957 (12.78)	0.1459	1955 (17.48)	1979 (17.69)	0.0055
HBp	4403 (37.23)	10777 (27.79)	0.1952	4036 (36.08)	4028 (36.01)	0.0015
ICU	627 (5.3)	570 (1.47)	0.171	399 (3.57)	477 (4.26)	0.0311
Surgical	7594 (64.2)	27759 (71.58)	0.1055	7311 (65.35)	7292 (65.18)	0.0027
Medical	3607 (30.5)	10454 (26.96)	0.08	3477 (31.08)	3418 (30.55)	0.0097
OP	7455 (63.03)	24692 (63.67)	0.0132	6897 (61.65)	7021 (62.76)	0.023
ACEI/ARB	2744 (23.2)	4626 (11.93)	0.267	2328 (20.81)	2390 (21.36)	0.0131
CCB	2382 (20.14)	4830 (12.45)	0.1916	2107(18.83)	2133 (19.07)	0.0058
Statin	3724 (31.48)	7610 (19.62)	0.2554	3264 (29.18)	3311 (29.6)	0.009
NASID	2442 (20.65)	9427 (24.31)	0.0904	2334 (20.86)	2360 (21.1)	0.0057
Antibiotic	8663 (73.24)	22043 (56.84)	0.3705	7966 (71.21)	8050 (71.96)	0.017

^a^*ASMD* absolute standardized mean difference

### Clinical characteristics of furosemide use in AKI patients

Based on the correlation analysis, predicting AKI using a single clinical characteristic is challenging, as no individual indicator can directly reflect the condition (Additional file 1: Fig S1). However, it is worth noting that ICU admission had the strongest correlation with AKI (*r* = 0.24, *P* < 0.05). Furosemide, an essential factor influencing AKI, had a correlation coefficient of 0.19 for single-day total dose, 0.16 for daily average dosage, and 0.15 for duration related to AKI. The AKI group had a higher single-day total dose, average daily dose, and cumulative dose of furosemide compared to the non-AKI group, alongside a longer duration of furosemide use (Table 2).

**Table 2:** Difference between the AKI and Non-AKI when using furosemide

Furosemide	First AKI during hospitalization	Non-AKI	Total
Number	2678	8509	11,187
Duration (day)	4.16 ± 5.1	3.5 ± 3.87	4.9 ± 6.28
Single-day total dose(mg)	58.02 ± 91.39	33.4 ± 34.79	46.26 ± 72.48
Total dose(mg)	194.06 ± 387.78	120.99 ± 233.5	209.59 ± 525.34
Average daily dose(mg/day)	41.53 ± 43.79	27.43 ± 20.13	31.74 ± 28.85

**Fig. 2:**
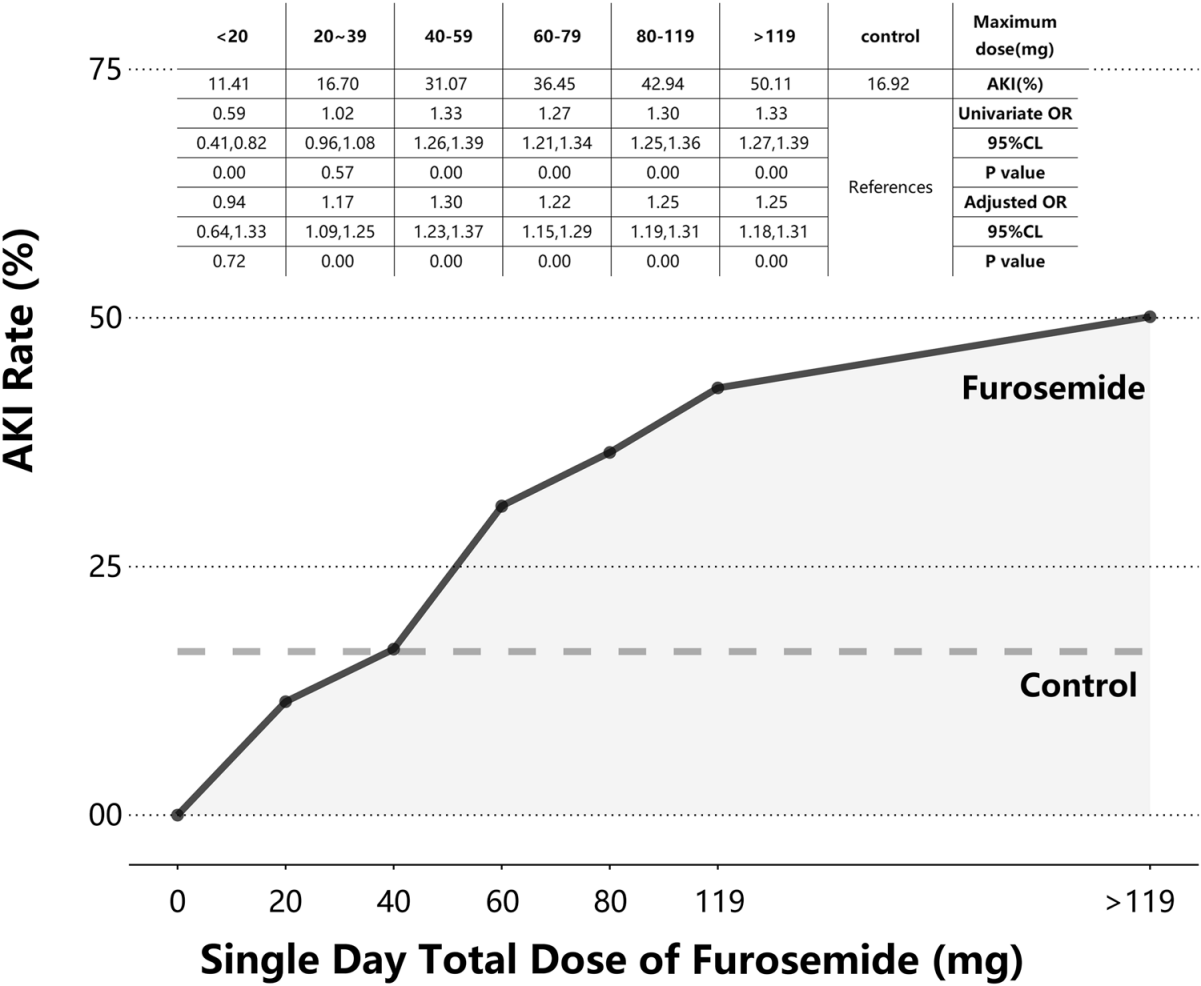
AKI rate in different maximum doses of furosemide

**Fig. 3:**
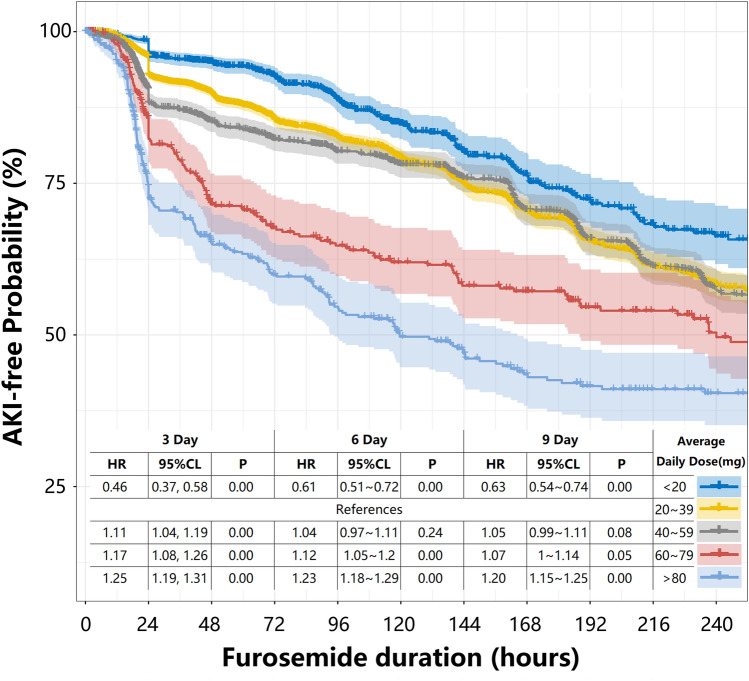
Stratification of non-AKI rate among patients in different daily doses of furosemide on the basis of COX multivariable adjustments

Since furosemide dosage has been found to be associated with the incidence of AKI, we conducted further investigations to determine whether the risk varied with different single-day total doses and cumulative total doses. First, when the single maximum dose of furosemide is less than 40 mg/day, the incidence of AKI is similar to the control group (16.72% vs. 16.92%). However, as the cumulative single-day total dose of furosemide increases, the risk of AKI also rises (Fig. 2). After adjusting for multi-factor regression, we found that 20 mg/day of furosemide did not increase the risk of AKI. Conversely, a dose of 20–39 mg/day of furosemide moderately increases the risk, while a dosage exceeding 40 mg/day significantly raises the risk compared to the control group. Second, we found that as the average daily dose of furosemide increases, the probability of AKI also increases. Within the first 3 days, the AKI response to the average daily dose of furosemide is similar to t the single-day total dose. In the subsequent 6 days, the AKI risk does not increase for doses of 40–59 mg/day compared to 20–39 mg/day. During the remaining duration, there is no significant difference between doses of 60–79 mg/day and 40–59 mg/day (Fig. 3). These findings indicate that the risk of AKI in response to furosemide doses ranging from 20 to 79 mg/day shows minimal variation during long-term use. In contrast, smaller doses are still necessary for short-term use to prevent AKI.

### Baseline characteristics of AKI patients with furosemide using or not

**Fig. 4:**
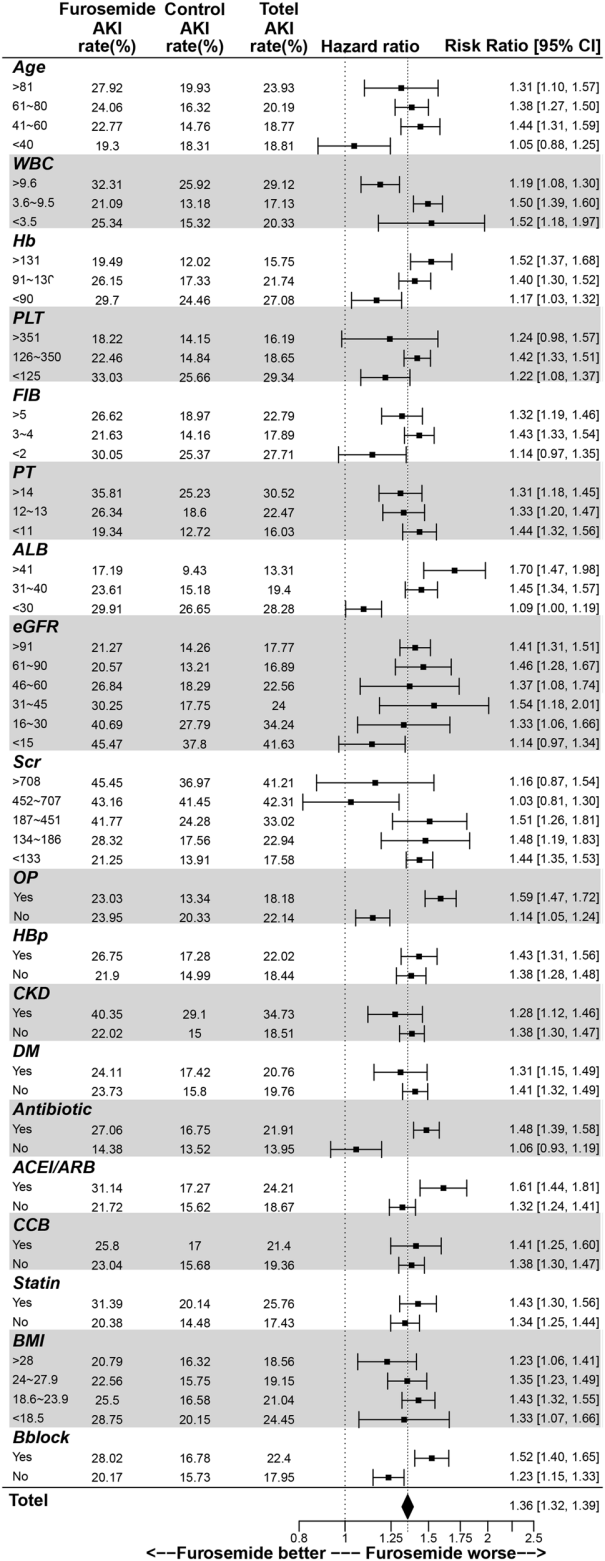
Forest plot of risk ratio of acute kidney injury for patients taking furosemide or not after propensity score matching

Subgroup analysis was conducted to investigate the risk of AKI associated with furosemide use in different clinical interventions or patient conditions (Fig. 4). Our findings revealed varying degrees of risk for AKI in response to furosemide based on patient age: the risk of AKI increases with both patient age and furosemide use, but furosemide does not affect the risk of AKI in patients less than 40 years (19.3% vs. 18.31, RR = 1.05, 95% CI 0.88 ~ 1.25). In addition, when other indicators are abnormal (e.g., Hb, white blood cells, PLT, plasma fibrinogen (PT), prothrombin time, Scr, eGFR, and albumin), the risk of AKI is higher, and furosemide contributes to moderate risk of AKI. Moreover, furosemide usage is closely associated with the risk of AKI, even in patients with normal clinical characteristics. Notably, furosemide may have a synergistic effect with factors related to hospitalization and concomitant drugs. Furosemide users who had undergone surgical operations (RR = 1.59, 95% CI 1.47–1.72), previous treatment with β-blockers (RR = 1.52, 95% CI 1.40–1.65) and ACEI/ARB (RR = 1.61, 95% CI 1.44–1.81), or combined with antibiotics (RR = 1.48, 95% CI 1.39–1.58) had a higher risk of AKI.

### Risk factors of AKI patients with furosemide administration

AKI associated with furosemide administration is a complex hospitalization-related disease influenced by multiple factors. Univariate analysis revealed that among inpatients, the ICU administration had the highest correlation with AKI (*r* = 0.24, *P* < 0.05), followed by the single-day total dose of furosemide (*r* = 0.20, *P* < 0.05), daily average dose (*r* = 0.13, *P* < 0.05), and approximately 15 other hospitalization-related variables (*r* > 0.13, *P* < 0.05) (Additional file 2: Fig S2).

To identify reliable predictors of furosemide-related AKI and eliminate multicollinearity, we conducted forward stepwise linear regression analyses. Among the variables considered, the single-day total dose of furosemide (β = 0.23, OR = 1.25, 95% CI 1.22–1.28, *P* < 0.001) emerged as the most critical variable in interpreting the regression function, suggesting a higher probability of furosemide-associated AKI with increasing dosage. The ICU setting (β = 4.46, OR = 0.83, 95% CI 3.58–5.57, *P* < 0.001) ranked second in importance and served as a prognostic factor, specifically among furosemide users (Table 2). Subsequently, the Scr level (β = 0.36, OR = 1.44, 95% CI 1.35–1.52) demonstrated the third-highest impact. At the same time, factors such as antibiotics, statin, NSAIDs, CKD, PT, β-blocker, aspartate transaminase, high-density lipoprotein, ACEI/ARB, PPI, PLT, BMI, and surgery made only marginal contributions to the risk of AKI compared to the furosemide dosage, ICU administration and Scr level (Table 2). Furthermore, a multicollinearity analysis was conducted to assess the linear relationships among the variables. It was observed that 15 variables displayed substantial nonlinearity (VIF < 2), establishing their appropriateness for constructing a logistic regression model (Table 3).

**Table 3:** Multivariable logistic regression model after forward selection of variables

Characteristics	Estimate	OR	2.50%	97.50%	AIC	P value
Furosemide	0.22119	1.247555	1.217872	1.27806872	11583	2.00E-16
ICU	1.44817	4.255333	3.404765	5.32816255	11983	2.00E-16
Scr	0.36473	1.44013	1.356409	1.52938821	12,015	2.00E-16
Antibiotic	0.79609	2.21685	1.961907	2.50931441	12025	2.00E-16
Statin	0.45986	1.583856	1.412764	1.77522011	12040	2.93E-15
NSAIDs	0.39432	1.483372	1.335555	1.64714551	12050	1.68E-13
TBIL	0.314	1.368888	1.271853	1.47325015	12080	2.00E-16
beta-blocker	0.29629	1.34486	1.213508	1.49031899	12084	1.58E-08
ACEI/ARB	0.39502	1.484418	1.313663	1.67672349	12092	2.21E-10
AST	0.35003	1.419117	1.312738	1.53453523	12093	2.00E-16
HDL	− 0.16944	0.844134	0.810078	0.87950752	12110	6.58E-16
PPI	0.74226	2.100686	1.769073	2.50528815	12148	2.00E-16
BMI	− 0.16873	0.844734	0.794016	0.89847073	12250	8.69E-08
Surgery	0.71072	2.03545	1.803569	2.29962969	12261	2.00E-16

### Prediction model of furosemide-related AKI

**Fig. 5:**
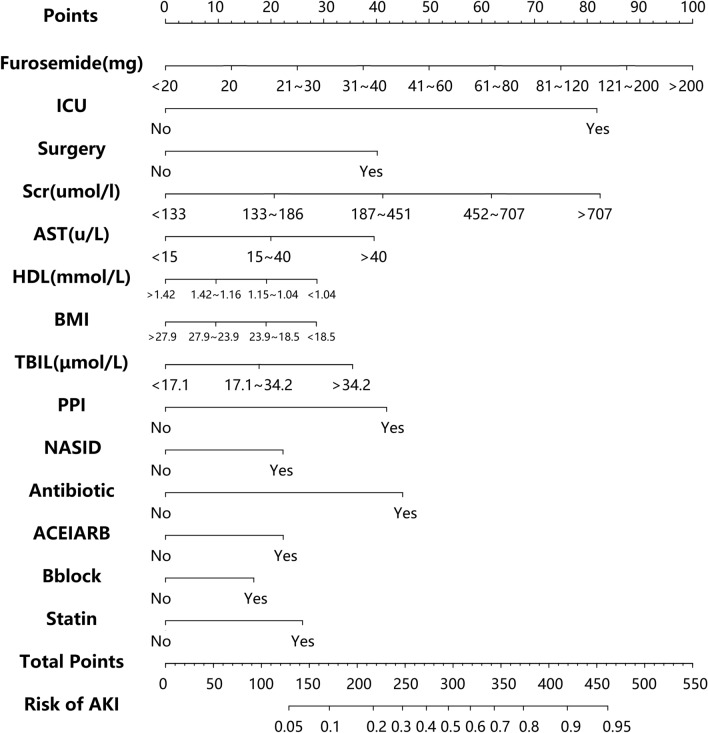
Nomogram to predict the probabilities of FM-AKI when using furosemide. For each patient administered furosemide, the points of the corresponding clinical parameters were calculated and summed up to obtain the total points. The predicted probabilities of FM-AKI can be estimated based on the total points

Based on forward stepwise regression analyses, we developed a logistic regression model to assess independent factors and estimate their effects on the occurrence of furosemide-related AKI in furosemide users. Subsequently, a nomogram was constructed to visualize the data and assign scores to each independent prognostic factor. As a result, the furosemide dosage exhibited the highest contribution, followed by ICU administration, Scr, antibiotics, statins, NSAIDs, β-blockers, AST, HDL, ACEI/ARB, proton pump inhibitors, BMI, and surgery, which had moderate impacts on the score. These findings were consistent with the stepwise regression. By summing up the scores along a straight line on the total point scale, we were able to calculate the total score and estimate the risk of furosemide-associated AKI. Patients with high total scores when using furosemide may have a high risk of AKI, while patients with lower total scores had a better chance of avoiding AKI (Fig. 5). The model discrimination analysis showed that the nomogram had a C-index of 0.7740, indicating that the nomogram correctly discriminated the outcome with a probability of 77.40%.

## Discussion

Our result found that furosemide, especially the single-day total dose, is an important factor affecting HA-AKI occurrence. Factors associated with susceptibility to HA-AKI in patients with furosemide usage included ICU administration and eGFR level, antibiotics, Statin, NSAIDs, CKD, PT, β-block, AST, HDL, ACEI/ARB, PPI, PLT, BMI, and surgery history. Renal function should be close monitored in furosemide usage at clinical practice.

The association of furosemide use with AKI remains controversial [27–30]. Theoretically, furosemide may prevent AKI by decreasing the GFR and tubular reabsorption and reducing renal medullary oxygenation [31–34]. In addition, some scholars assumed that furosemide could act as renal vasodilators thus preventing AKI [35]. However, in our study, using a more rigorous analytic approach and inclusion/exclusion criteria, 11187 and 11187 consecutive inpatients taking or not taking furosemide were enrolled in the final cohort. We found that whether in univariate, multivariate, or stepwise regression, and in intra-group or groups comparison, furosemide is an important factor affecting AKI. In accordance with our findings, several newly published studies depicted furosemide's ability to exacerbate as well as increase the risk of AKI occurrence: a meta-analysis published by Ho and Sheridan included 9 studies (3 on the prevention of AKI and the remaining 6 on the treatment of AKI) showed that furosemide has no obvious clinical benefits in the prevention and treatment of AKI in adult patients. Even worse, furosemide treatment may prolong hospital stay, and high doses of furosemide have a short-term risk of deafness or tinnitus, which is detrimental to patients in the ICU department [36]. Another study also showed that furosemide administration had no effect on mortality or RRT demand regardless of intervention strategy, AKI etiology, control drug and furosemide dose [14, 36–38]. These findings are biologically plausible due to various causes like activation of the renin–angiotensin system or the sympathetic nervous system and a relative decrease in medullary blood flow compared to the cortex with furosemide treatment [39]. Therefore, it is necessary to pay attention to renal function during furosemide management.

Our findings added to the limited data on the association of furosemide dosage and hospitalized-acquired AKI in general populations. In line with our findings, a substantial cohort from EACH study similarly observed an elevated incidence of HA-AKI following furosemide administration [39]. By providing a response curve of furosemide with the HA-AKI risk, they found a linear, positive association between cumulative dose, maximum daily dose, cumulative usage days and HA-AKI. However, their study did not provide a specific dosage recommendation. In the present study, we identified that the total dose of furosemide administered within a single day plays a crucial role in the development of AKI. Specifically, a dose of 20 mg/day did not elevate the risk of AKI, whereas exceeding 40 mg/day significantly increased the risk compared to the control group. Furthermore, our findings revealed a positive association between the average daily dose of furosemide and the probability of AKI occurrence. Therefore, since the metabolism of furosemide is affected by kidney function, more attention should be paid to observing renal function in clinical use [40]. Moreover, we also found that during the initial 3 days, the response of AKI to the average daily dose mirrored that of the single-day total dose. Notably, the risk of AKI associated with furosemide doses ranging from 20 to 79 mg/day showed minimal variation with prolonged usage. This may be attributed by that when diuretics are administered for treatment, drugs that promote sodium excretion can result in a negative sodium balance. This reduction in extracellular fluid prompts a compensatory response to maintain homeostasis, wherein sodium retention in the renal tubules is increased by activating the renin–angiotensin–aldosterone and sympathetic nervous systems. This response does not specifically target the diuretics themselves. Over a few days, this steady-state response establishes a new equilibrium compatible with the lowered extracellular fluid state. This steady-state response corresponds to the decreased blood flow encountered during subsequent diuretic therapy. In patients with heart failure, secondary aldosterone levels rise, leading to pronounced and rapid reabsorption of sodium, which consequently contributes to diuretic resistance [41]. Therefore, patients with a high risk of AKI are more prone to diuretic resistance. Thus, patients may need to change or combine other diuretics for diuresis. However, though these findings necessitate further investigation through larger-scale studies involving diverse populations, we still strongly advise clinicians to exercise increased vigilance regarding the potential occurrence of AKI following furosemide administration, particularly in patients receiving treatment with high doses.

Patients with varying clinical settings should receive tailored treatment when prescribed furosemide, given its high propensity for drug–drug interactions [42]. Our investigation revealed that the coadministration of furosemide with certain agents increases the risk of AKI, with antibiotics being the most implicated, followed by ACEI/ARB and β-blocker. Antibiotics, such as penicillin, are vital in clinical practice but can competitively interact with furosemide due to their organic acid properties. This interaction intensifies antibiotics' nephrotoxic effects and diminishes furosemide’s diuretic efficacy of furosemide [43]. Consistent with earlier studies, other drugs antihypertensive drugs (e.g., ACEI/ARB, β-blockers) also increase the risk of AKI, likely attributable to reduced renal perfusion and intraglomerular pressure, ultimately leading to AKI [44]. In addition, we identified surgery as a risk factor for furosemide-associated AKI, primarily because furosemide administration may exacerbate intravascular volume contraction, particularly during the postoperative period when fluids shift from the intravascular space to the third space [45].

Recently, studies have highlighted the occurrence of “moderate deteriorations in renal function commonly encountered with aggressive diuresis.” Interestingly, the decline in eGFR resulting from aggressive diuresis is believed to be caused by unknown mechanisms rather than renal tubular injury [46, 47]. Notably, these findings were obtained in an ideal situation, specifically in the context of heart failure. Considering the widespread use of furosemide in various diseases and the complexities of real clinical settings, it is crucial to address the multi-factorial nature of AKI. Case–control studies that strictly control entry criteria may not fully capture the actual effects of furosemide. To account for the diverse clinical scenarios encountered in real-life practice, we analyzed various factors contributing to AKI while controlling for confounding variables through score matching and multiple factor adjustments. Subsequently, we developed a prognostic nomogram that provides visualized predictions for the outcomes of furosemide users [48, 49]. Our findings indicate that patients with higher nomogram scores may have an increased risk of AKI. These results were further validated through calibration analyses, demonstrating an excellent agreement between nomogram predictions and the actual occurrence of AKI, with a probability of 77.40%. Consequently, our model exhibits high accuracy and holds promise for clinical utilization.

There are several limitations in this study. First, this was a single-center study that needs future studies to validate our results in a broader setting. Second, AKI is a very complex disease and difficult to predict. Although we collected nearly all comprehensive indicators and used reasonable statistical methods to eliminate bias, there may still be potential factors being omitted that could lead to AKI. Third, variables with more than 15% missing values were not included in this analysis, this may affect the results. COX and Logistic regression models are reliable classification and prediction methods, but these approaches are not the best analytical methods. Considering the rapid development of machine learning, which may help us attain better prediction results, the international medical community needs to gradually recognize more advanced methods as superior [50, 51]. In addition, although Ng KT et al. reported that continuous furosemide infusion is more effective in achieving sustained diuresis compared to intermittent bolus therapy [52], Michael Joannidis et al. have highlighted the lack of evidence supporting better prognosis with continuous administration over bolus administration [30]. Furthermore, there is limited research conducted on the application mode of furosemide and its association with AKI development in the general population. Therefore, further investigation is warranted. Finally, the etiology of AKI was not distinguished in the current study, and patients with hypervolemia were also included, which is necessary to validate the results through additional research.

In conclusion, using furosemide in clinical practice requires discretion, especially in its dosage, combination with other medications and the renal function of patients. Our prognostic model can predict the probability of furosemide-related AKI after using furosemide accurately. Additional studies are required to determine whether this prognostic model can be applied to other institutions or countries.

### Supplementary Information

**Additional file 1: **Correlation analysis of AKI patients with furosemide administration.

**Additional file 2: **Univariate analysis of AKI patients with furosemide administration.

## Data Availability

The data sets generated during and/or analyzed are available from the corresponding authors upon request.
