# Supplementary figures and images for: Hospitalized patients received furosemide undergoing acute kidney injury: the risk and prediction tool
Source: Eur J Med Res. 2023 Sep 2;28:312. doi: 10.1186/s40001-023-01306-0 (PMC10474726; doi:10.1186/s40001-023-01306-0)

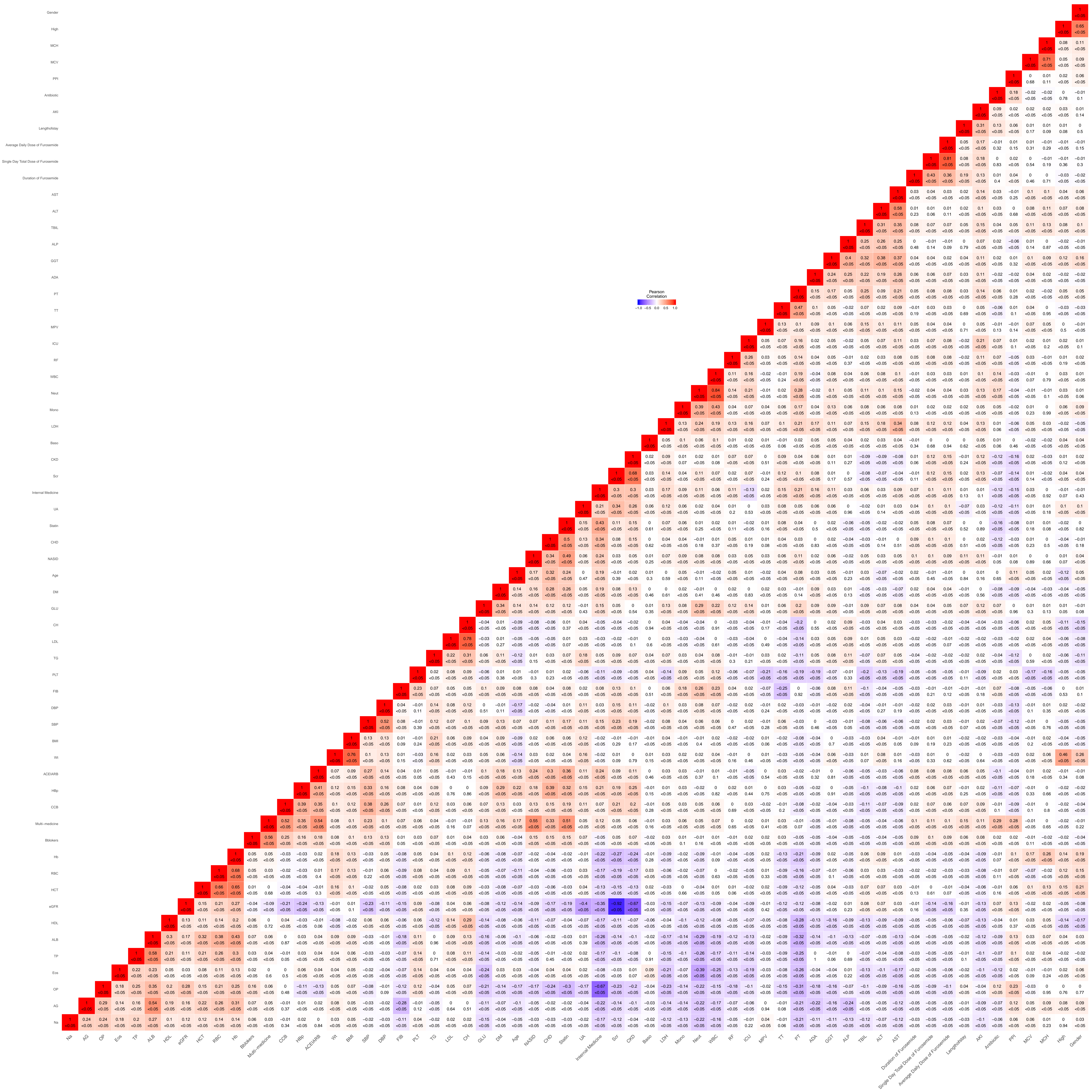

Supplement: Supplementary file 1 — Additional file 1: Correlation analysis of AKI patients with furosemide administration. [file 40001_2023_1306_MOESM1_ESM.pdf]

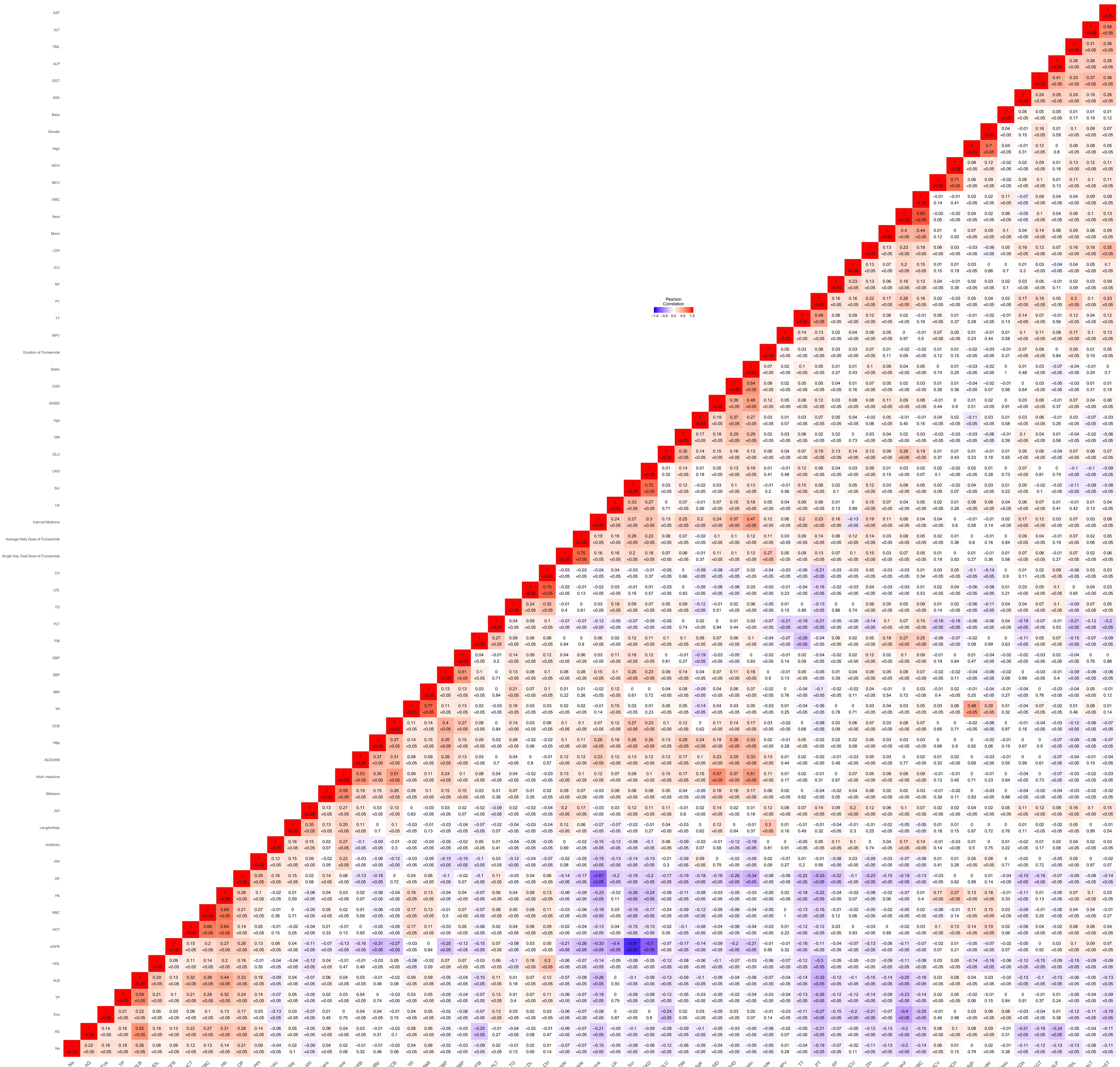

Supplement: Supplementary file 2 — Additional file 2: Univariate analysis of AKI patients with furosemide administration. [file 40001_2023_1306_MOESM2_ESM.pdf]
